# Association between planetary health diet index and lung cancer risk in 106,542 participants: a prospective cohort study

**DOI:** 10.3389/fnut.2026.1794585

**Published:** 2026-04-29

**Authors:** Junjin Zhu, Haitao Gu, Ziyao Zeng, Yi Xiao, Yangpiaoyi Shi, Zhilin Me, Linglong Peng, Ling Xiang, Yuxiang Luo, Yikuan Chen, Xuemei Jiang

**Affiliations:** 1 Department of Gastrointestinal Surgery, The Second Affiliated Hospital of Chongqing Medical University, Chongqing, China; 2 Department of Clinical Nutrition, The Second Affiliated Hospital of Chongqing Medical University, Chongqing, China; 3 Erasmus University Medical Center, Rotterdam, Netherlands; 4 Department of Vascular Surgery, The Second Affiliated Hospital of Chongqing Medical University, Chongqing, China

**Keywords:** cancer prevention, epidemiology, lung cancer, planetary health diet, planetary health diet index

## Abstract

**Background:**

The Planetary Health Diet Index (PHDI) is a validated tool for assessing adherence to the Planetary Health Diet (PHD)—a dietary pattern designed to address both human wellness and environmental sustainability. However, limited studies have specifically investigated the association between PHDI and lung cancer susceptibility—a leading global cancer mortality cause. Our study intends to explore this potential association.

**Methods:**

Data for this analysis were derived from the prospective cohort of the Prostate, Lung, Colorectal, and Ovarian (PLCO) Cancer Screening Trial. The PHDI was calculated for each participant to quantify their adherence to the target dietary pattern. Cox proportional hazards regression models were applied to estimate the hazard ratios (HRs) and 95% confidence intervals (CIs) for the association between PHDI and the incidence of lung cancer and its distinct subtypes. Subgroup analyses were conducted to identify potential effect modifiers that might influence the observed association, while sensitivity analyses were performed to verify the robustness and stability of the study findings.

**Results:**

Over a follow-up period of 8.8 years, 1,846 lung cancer cases were identified among 106,542 participants. In the fully adjusted model, participants in the highest quartile (Quartile 4) of the PHDI had a 29% lower lung cancer risk compared to those in the lowest quartile (Quartile 1) (HR _Quartile 4 vs Quartile 1_: 0.71, 95% CI: 0.62, 0.81; P _trend_ < 0.001). This inverse association was consistent across both non-small cell lung cancer (NSCLC) and small cell lung cancer (SCLC). Restricted cubic spline plots revealed a non-linear inverse dose–response relationship between PHDI and lung cancer risk (P _non-linear_: 0.043) as well as NSCLC risk (P _non-linear_: 0.035), whereas a linear inverse association was observed for SCLC (P _non-linear_: 0.956). No significant effect modification was detected in subgroup analyses, and the core findings remained stable and reliable following multiple sensitivity analyses.

**Conclusion:**

Our findings suggest that higher PHDI is inversely associated with lung cancer risk and its subtypes (NSCLC and SCLC), supporting the potential of PHD as a dietary strategy for lung cancer prevention.

## 1 Introduction

According to the Cancer Statistics, 2025 published in CA: A Cancer Journal for Clinicians, lung cancer-related deaths in the United States are projected to account for approximately 20% of all cancer deaths in 2025, a proportion that far surpasses that of any other malignant neoplasm (1). Hence, a substantial body of epidemiological studies has been devoted to elucidating the links between lung cancer and modifiable risk factors. Specifically, many previous studies have demonstrated that the occurrence of lung cancer is related to a variety of dietary factors: for example, high fruit and vegetable intake is linked to reduced risk (2–6). However, merely considering a single factor often neglects the complex interaction among various dietary factors.

The EAT-Lancet Commission recently introduced a novel nutritional framework termed the “Planetary Health Diet (PHD)”, designed to optimize both human and planetary health (7). Given its emphasis on overall dietary balance and benefits to both human beings and environment, along with its clinical applicability, investigating the relationship between this dietary pattern and lung cancer susceptibility is crucial for informing prevention efforts. PHD emphasizes high intake of plant-based foods and low intake of processed meats, which may modulate lung carcinogenesis via antioxidant and anti-inflammatory effects. To date, consensus on this association remains elusive: two prospective cohort studies conducted within the large UK Biobank population have reported inconsistent findings. One prospective cohort study reported an inverse relationship between PHD adherence and lung cancer incidence (8), while another observed sex-specific effects—this inverse link was restricted to men, with no significant association in women (9). These inconsistent results highlight the need for further validation. Accordingly, our study utilized the PHDI, a validated, energy-adjusted scoring tool for quantifying PHD adherence (10), to elucidate the relationship between this dietary pattern and lung cancer risk, thereby informing potential strategies for incidence reduction.

## 2 Methods

### 2.1 Study population

The data for this study were sourced from the Prostate, Lung, Colorectal, and Ovarian (PLCO) Cancer Screening Trial, a large-scale, multicenter randomized controlled trial (RCT) whose primary objective was to evaluate whether targeted screening modalities reduce mortality from these malignancies. The database contains data from 154,887 participants (with no prior history of these cancers and no recent screening for these malignancies), who were enrolled from November 1993 to September 2001 with the age between 55 and 74 years. Participants were then randomly assigned to two arms: the intervention arm received predefined cancer screening panel from PLCO, while the control arm received routine clinical care from their primary care providers. After randomization, several structured questionnaires were distributed to collect comprehensive information for each participant. The Baseline Questionnaire (BQ) collected the information of participants’ demographics, personal and family cancer history, smoking habits and other lifestyle factors at the beginning of the trial. The Diet History Questionnaire (DHQ) collected the information of participants’ usual dietary intake and drinking habits over a specific period.

**Figure 1:**
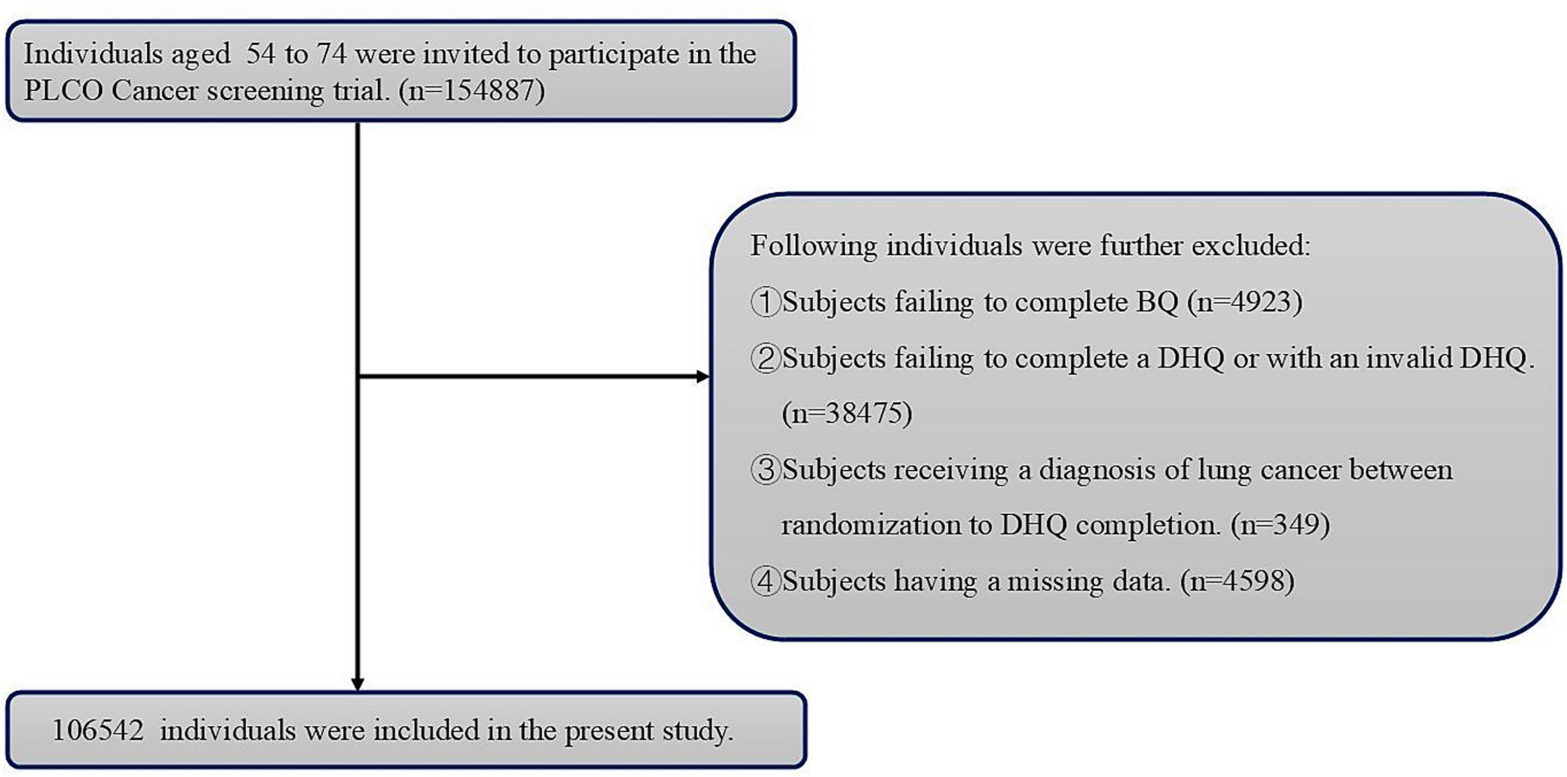
The flow chart of identifying eligible subjects. PLCO: Prostate, Lung, Colorectal, and Ovarian; BQ: Baseline questionnaire; DHQ: Diet history questionnaire.

**Figure 2:**
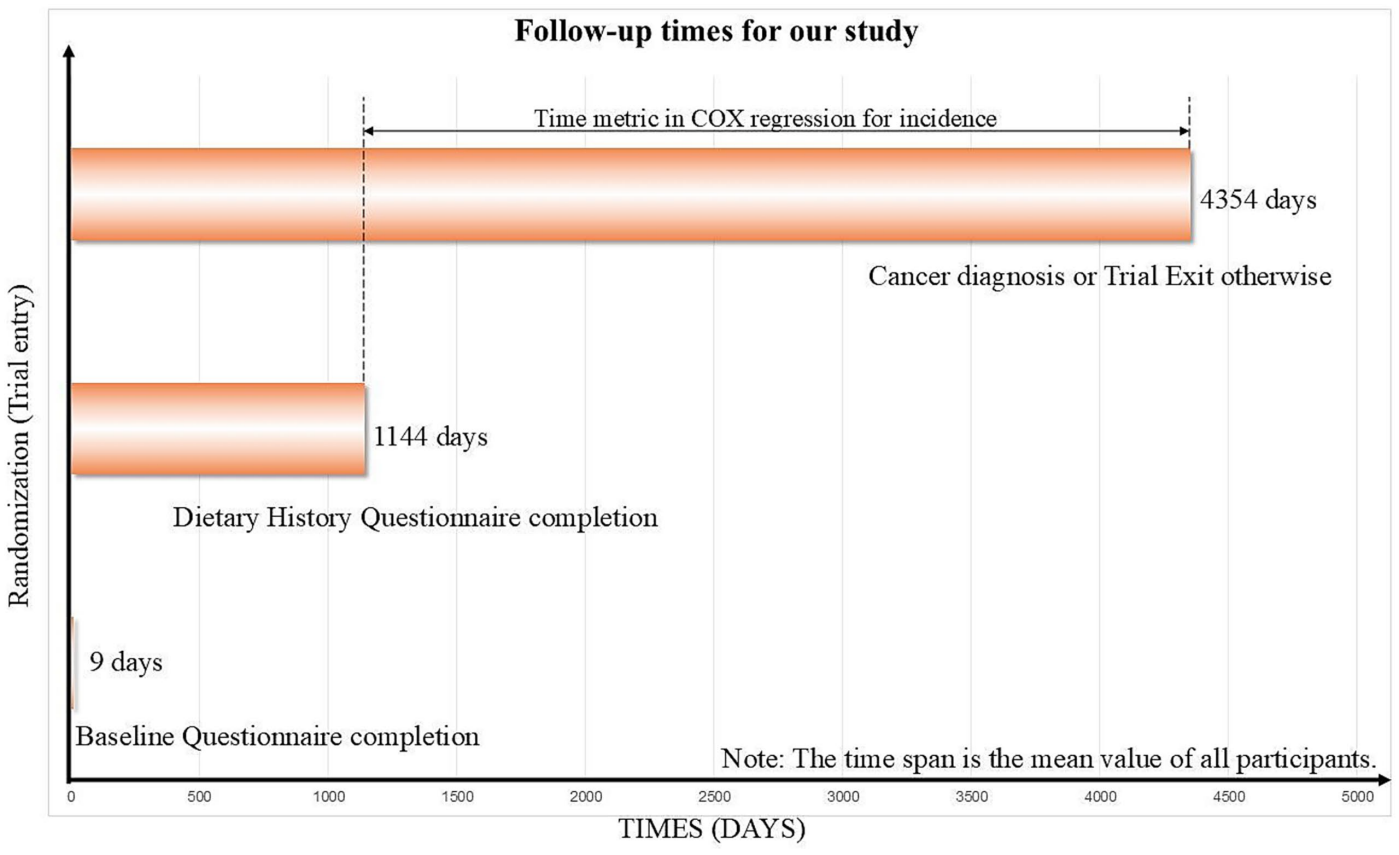
The timeline and follow-up scheme of our study.

In order to be objective, we excluded participants with such characteristics (Figure 1): (1) participants who did not complete BQ (*n* = 4,923); (2) participants who did not complete DHQ or with an invalid DHQ (*n* = 38,475); (3) participants who were diagnosed with lung cancer between randomization to DHQ completion (*n* = 349); (4) participants who had any missing data relevant to this study (*n* = 4,598). After exclusions, 106,542 participants were enrolled. The follow-up duration, an average of 8.8 years for each participant was calculated from the date of DHQ completion until the exit times (Figure 2). Exit dates were defined as the day of lung cancer diagnosis or the day last known lung cancer free.

### 2.2 Assessment of PHDI

The PHDI was developed to evaluate the individuals’ adherence to PHD in a way of using energy content. With data from the DHQ, we derived the consumption levels for each component of the PHDI and converted the unit of measurement from grams to calories using the United States Department of Agriculture (USDA) food energy conversion factors. Then, PHDI was calculated following the method proposed by Leandro Teixeira Cacau in 2021 without modification (10). A clear scoring rule is on the Supplementary Table 1. Overall, the 16 scoring criteria have been classified into four major categories: adequate component (nuts and peanuts, legumes, fruits, vegetables, whole grains), optimal component (eggs, fish and seafood, potatoes, dairy, vegetable oils), ratio component (dark green to total vegetables ratio, red and orange to total vegetables ratio) and moderation component (red meat, chicken, animal fats, added sugars). With the exception of the components in the ratio component, which employ a scale of 0 to 5, all remaining components are rated on a 0 to 10 scale. The final PHDI was the sum of each component’s score. Higher PHDI values indicated greater compliance with the PHD.

### 2.3 Determination of lung cancer

In our study, participants were required to fill out a form every year. In this form, they were asked to indicate whether they had been diagnosed with lung cancer and to provide relevant information about the cancer, including, but not limited to, self-reported information, family-provided reports, and official certificates. The histopathologic type of lung cancer was derived from ICD-O-2 morphology. If participants did not respond to the form, our researchers would contact them again by phone or email to confirm their situation.

### 2.4 Assessment of covariates

Taking into account possible confounding factors, BQ and DHQ were used to collect information on age, gender, race, education level, marital status, family history of lung cancer, body mass index (BMI), smoking status, alcohol drinking status, emphysema history, chronic bronchitis history, history of hypertension, aspirin use, and history of diabetes.

### 2.5 Statistical analysis

In our study, the lung cancer risk was measured in terms of incidence and its association with PHDI was described by Cox proportional hazards model. The study population was grouped into quartiles according to their PHDI levels, with the lowest quartile (Quartile 1) designated as the reference group. We calculated incidence rates, hazard ratios (HRs), and 95% confidence intervals (CIs) for each quartile, and then compared reference group with other three groups in the following three sequentially adjusted models. Unadjusted model: No covariates were included. Model 1: Adjusted for demographic factors, including age, sex, race, education levels, marital status. Model 2: Further adjusted for clinical and lifestyle covariates on top of Model 1, including family history of lung cancer, baseline BMI, smoking status, alcohol drinking status, emphysema history, chronic bronchitis history, hypertension history, diabetes history, aspirin use. The covariates incorporated in the aforementioned model were not arbitrarily selected according to subjective preferences. Instead, they were meticulously chosen based on clinical expertise and existing academic literature (11–13), where they had been identified as statistically significant covariates. We further delved into the relationship between PHDI and the incidence of lung cancer subtypes (SCLC and NSCLC). Subsequently, for each participant group, the median PHDI score was used as a representative value, treating it as a continuous variable in statistical models. By this approach, *p*-values for trend (P _trend_) were calculated, thereby allowing assessment of a consistent directional association between PHDI and lung cancer risk and determination of the underlying trend. To visually illustrate the risk of lung cancer and the dose–response trend across the full range of PHDI values, restricted cubic spline plots were utilized. Additionally, *p* value of nonlinearity (P _non-linear_) was computed to determine whether a nonlinear dose–response relationship exists between lung cancer risk and the PHDI. Furthermore, to elucidate the distinct contribution of each component and clarify the key determinants, we separately analyzed the relationship between each of the four PHDI component scores and lung cancer risk.

We carried out prespecified subgroup analyses according to multiple factors, including age (≤65 years or >65 years), sex (male or female), race (white or non-white), educational level (college below, some college and college graduate or postgraduate), marital status (married or unmarried), body mass index (≤25 or >25 kg/m^2^), smoking status (never or current/former), alcohol drinking status (never, current/former or unknown), family history of lung cancer (no, yes or unknown), history of emphysema (no or yes), history of chronic bronchitis (no or yes), history of diabetes (no or yes), and aspirin use (no or yes). The statistical significance of interactions was ascertained by calculating the *p*_value_ of the likelihood ratio test. Finally, several sensitivity analyses were performed to verify the robustness of the research findings: (1) excluding participants with emphysema history; (2) excluding participants with chronic bronchitis history; (3) excluding participants with extreme BMI (top 1% or bottom 1% in the included population); (4) excluding extreme energy intake (>4,000 kcal/day or <500 kcal/day); (5) excluding cases observed within the first 1 year; (6) excluding cases observed within the first 2 years; (7) excluding participants with family history of lung cancer.

Data analysis was conducted through R statistical package (version 4.5.1). The investigation employed a two-sided significance level of 0.05 to determine the statistical relevance of research findings.

## 3 Results

### 3.1 Participant characteristics

After elimination, 106,542 qualified participants were registered, including 50,993 (47.86%) males and 55,549 (52.14%) females with a mean (standard deviation) PHDI of 63.71 (12.56). Study population was stratified into four quartiles according to their PHDI values: Quartile 1, PHDI < 55.10; Quartile 2, PHDI 55.10–63.52; Quartile 3, PHDI 63.53–72.09; Quartile 4, PHDI > 72.09. The higher the PHDI, the greater the adherence to the PHD.

Demographically, in comparison to Quartile 1 (PHDI <55.10), participants in Quartile 4 (PHDI >72.09) tended to be older, female, non-white, unmarried, and having higher educational attainment. Behaviorally, compared to Quartile 1, Quartile 4 demonstrated a significantly higher probability of being lifelong non-smokers and non-drinkers, coupled with fewer smoking pack-years, a lower BMI, and reduced daily energy intake. All detailed baseline characteristics are presented in Table 1.

**Table 1:** Baseline characteristics of study population according to quartiles of planetary health diet index*.

Characteristics	Overall	Quartiles of planetary health diet index			
		Quartile 1 (≤55.09)	Quartile 2 (55.10–63.52)	Quartile 3 (63.53–72.09)	Quartile 4 (>72.09)
Number of participants	106,542	26,630	26,653	26,624	26,635
Planetary Health Diet Index	63.71 ± 12.56	47.90 ± 5.79	59.45 ± 2.42	67.65 ± 2.46	79.85 ± 6.47
Age	62.49 ± 5.30	61.93 ± 5.22	62.45 ± 5.28	62.72 ± 5.32	62.85 ± 5.32
Age level					
≤59	36,017 (33.81%)	10,219 (38.37%)	9,023 (33.85%)	8,508 (31.96%)	8,267 (31.04%)
60–64	33,150 (31.11%)	8,106 (30.44%)	8,399 (31.51%)	8,336 (31.31%)	8,309 (31.20%)
65–69	23,935 (22.47%)	5,498 (20.65%)	5,918 (22.20%)	6,152 (23.11%)	6,367 (23.90%)
≥70	13,440 (12.61%)	2,807 (10.54%)	3,313 (12.43%)	3,628 (13.63%)	3,692 (13.86%)
Sex					
Male	50,993 (47.86%)	14,173 (53.22%)	12,655 (47.48%)	12,071 (45.34%)	12,094 (45.41%)
Female	55,549 (52.14%)	12,457 (46.78%)	13,998 (52.52%)	14,553 (54.66%)	14,541 (54.59%)
Race					
White	97,141 (91.18%)	25,173 (94.53%)	24,857 (93.26%)	24,271 (91.16%)	22,840 (85.75%)
Non-white	9,401 (8.82%)	1,457 (5.47%)	1796 (6.74%)	2,353 (8.84%)	3,795 (14.25%)
Education level					
College below	44,857 (42.10%)	13,023 (48.90%)	11,751 (44.09%)	10,732 (40.31%)	9,351 (35.11%)
Some college and college graduate	41,732 (39.17%)	9,927 (37.28%)	10,367 (38.90%)	10,642 (39.97%)	10,796 (40.53%)
Postgraduate	19,953 (18.73%)	3,680 (13.82%)	4,535 (17.01%)	5,250 (19.72%)	6,488 (24.36%)
Marital status					
Married or living as married	83,450 (78.33%)	21,175 (79.52%)	21,194 (79.52%)	20,896 (78.49%)	20,185 (75.78%)
Other	23,092 (21.67%)	5,455 (20.48%)	5,459 (20.48%)	5,728 (21.51%)	6,450 (24.22%)
Body mass index (kg/m^2^)	27.21 ± 4.81	27.89 ± 4.95	27.47 ± 4.87	27.04 ± 4.69	26.44 ± 4.63
Smoking status					
Never	51,319 (48.17%)	11,493 (43.16%)	12,922 (48.48%)	13,241 (49.73%)	13,663 (51.30%)
Current	9,901 (9.29%)	3,906 (14.67%)	2,592 (9.72%)	1978 (7.43%)	1,425 (5.35%)
Former	45,322 (42.54%)	11,231 (42.17%)	11,139 (41.79%)	11,405 (42.84%)	11,547 (43.35%)
Smoking pack-years	17.94 ± 26.87	22.79 ± 30.46	18.22 ± 27.25	16.36 ± 25.25	14.41 ± 23.26
Alcohol drinking status					
Never	10,817 (10.15%)	2,565 (9.63%)	2,718 (10.20%)	2,783 (10.45%)	2,751 (10.33%)
Former	15,539 (14.58%)	4,205 (15.79%)	3,785 (14.20%)	3,721 (13.98%)	3,828 (14.37%)
Current	77,182 (72.44%)	19,151 (71.92%)	19,421 (72.87%)	19,364 (72.73%)	19,246 (72.26%)
Unknown	3,004 (2.82%)	709 (2.66%)	729 (2.74%)	756 (2.84%)	810 (3.04%)
Aspirin consumption					
No	56,638 (53.16%)	14,169 (53.21%)	14,129 (53.01%)	14,142 (53.12%)	14,198 (53.31%)
Yes	49,904 (46.84%)	12,461 (46.79%)	12,524 (46.99%)	12,482 (46.88%)	12,437 (46.69%)
History of diabetes					
No	99,404 (93.30%)	25,050 (94.07%)	24,921 (93.50%)	24,786 (93.10%)	24,647 (92.54%)
Yes	7,138 (6.70%)	1,580 (5.93%)	1732 (6.50%)	1838 (6.90%)	1988 (7.46%)
History of hypertension					
No	71,739 (67.33%)	17,990 (67.56%)	17,720 (66.48%)	17,904 (67.25%)	18,125 (68.05%)
Yes	34,803 (32.67%)	8,640 (32.44%)	8,933 (33.52%)	8,720 (32.75%)	8,510 (31.95%)
Family history of lung cancer					
No	92,849 (87.15%)	22,964 (86.23%)	23,205 (87.06%)	23,253 (87.34%)	23,427 (87.96%)
Yes	11,151 (10.47%)	2,906 (10.91%)	2,789 (10.46%)	2,773 (10.42%)	2,683 (10.07%)
Unknown	2,542 (2.39%)	760 (2.85%)	659 (2.47%)	598 (2.25%)	525 (1.97%)
Emphysema history					
No	104,295 (97.89%)	25,869 (97.14%)	26,074 (97.83%)	26,119 (98.10%)	26,233 (98.49%)
Yes	2,247 (2.11%)	761 (2.86%)	579 (2.17%)	505 (1.90%)	402 (1.51%)
Chronic bronchitis history					
No	101,969 (95.71%)	25,339 (95.15%)	25,477 (95.59%)	25,498 (95.77%)	25,655 (96.32%)
Yes	4,573 (4.29%)	1,291 (4.85%)	1,176 (4.41%)	1,126 (4.23%)	980 (3.68%)
Energy intake from diet (kcal/day)	1734.47 ± 731.91	1901.48 ± 800.10	1761.48 ± 739.30	1669.45 ± 690.57	1605.44 ± 655.53
Adequacy component					
Nuts and peanuts (kcal/day)	39.97 ± 85.96	30.82 ± 74.67	35.34 ± 80.17	39.90 ± 84.58	53.82 ± 100.53
Legumes (kcal/day)	45.04 ± 63.84	32.22 ± 38.89	38.84 ± 50.70	43.74 ± 55.95	65.34 ± 92.05
Fruits (kcal/day)	112.47 ± 95.06	68.69 ± 65.49	104.02 ± 85.07	123.97 ± 91.96	153.20 ± 111.30
Vegetables (kcal/day)	73.95 ± 48.38	64.55 ± 40.99	70.96 ± 44.63	74.56 ± 47.54	85.70 ± 56.53
Whole cereals (kcal/day)	214.33 ± 209.69	136.63 ± 138.92	186.23 ± 175.59	224.50 ± 201.49	309.98 ± 262.84
Optimum component					
Eggs (kcal/day)	15.69 ± 16.43	22.10 ± 22.02	15.93 ± 15.67	13.45 ± 13.15	11.26 ± 10.51
Fish and seafood (kcal/day)	22.10 ± 26.50	18.93 ± 26.87	21.27 ± 26.26	22.92 ± 25.61	25.29 ± 26.85
Tubers and potatoes (kcal/day)	45.44 ± 37.20	59.22 ± 42.82	49.48 ± 37.64	40.97 ± 33.44	32.07 ± 27.58
Dairy (kcal/day)	210.47 ± 173.98	245.71 ± 198.79	219.93 ± 175.56	203.10 ± 162.69	173.13 ± 146.61
Vegetable oils (kcal/day)	353.80 ± 193.82	427.49 ± 215.79	363.77 ± 191.19	326.27 ± 176.17	297.69 ± 163.32
Ratio component					
Dark green to total vegetables ratio	0.07 ± 0.07	0.05 ± 0.05	0.06 ± 0.06	0.08 ± 0.07	0.10 ± 0.09
Red and orange to total vegetables ratio	0.08 ± 0.06	0.06 ± 0.05	0.08 ± 0.06	0.09 ± 0.07	0.10 ± 0.08
Moderation component					
Red meat (kcal/day)	95.22 ± 80.09	121.58 ± 94.14	101.84 ± 80.57	87.81 ± 70.99	69.63 ± 61.75
Chicken and substitutes (kcal/day)	46.61 ± 48.37	51.45 ± 50.89	48.08 ± 49.91	45.79 ± 47.18	41.13 ± 44.70
Animal fats (kcal/day)	28.31 ± 46.68	52.68 ± 63.40	32.21 ± 45.70	19.39 ± 33.64	8.95 ± 20.30
Added sugars (kcal/day)	97.72 ± 95.82	131.24 ± 111.63	107.04 ± 95.43	88.97 ± 85.77	63.63 ± 73.19

*Values are mean ± standard deviation or counts (percentage) as indicated.

### 3.2 Association between PHDI and the risk of lung cancer

During an average follow-up period of 8.8 years, a total of 1846 cases of lung cancer were identified, including 1,586 non-small cell lung cancer (NSCLC) and 260 small cell lung cancer (SCLC). The overall incidence rate of lung cancer was 0.197 cases per 100 person-years. And its subtypes (NSCLC and SCLC) had incidence rates of 0.169 and 0.028 cases per 100 person-year, respectively. In the unadjusted Cox proportional hazards model, participants in the highest quartile exhibited a significantly reduced risk of lung cancer compared to those in the lowest quartile (HR _Quartile 4 vs Quartile 1_: 0.61; 95% CI: 0.54, 0.69; P _trend_ < 0.001). After adjustment for demographic covariates (Table 2, Model 1), this inverse association remained consistent (HR _Quartile 4 vs Quartile 1_: 0.65; 95% CI: 0.57, 0.74; P _trend_ < 0.001). Further adjustment for a comprehensive set of potential confounders (Table 2, Model 2) did not substantially alter the observed relationship (HR _Quartile 4 vs Quartile 1_: 0.71; 95% CI: 0.62, 0.81; P _trend_ < 0.001). Stratified analysis by lung cancer subtype revealed similar inverse associations for both NSCLC and SCLC. For NSCLC, participants in Quartile 4 had a significantly lower risk relative to Quartile 1 (HR _Quartile 4 vs Quartile 1_: 0.73; 95% CI: 0.63, 0.84; P _trend_ < 0.001). A comparable pattern was observed for SCLC (HR _Quartile 4 vs Quartile 1_: 0.59; 95% CI: 0.40, 0.85; P _trend_ < 0.001; Table 2).

**Table 2:** Hazard ratios and 95% CIs of lung cancer in the PLCO cohort, by quartiles of planetary health diet index.

Model	Planetary health diet index, HR (95% CI)				1-point increment in PHDI	*p* for trend ^a^
	Quartile 1 (lowest)	Quartile 2	Quartile 3	Quartile 4 (highest)		
Lung cancer^b^						
Cases	605	476	385	380		
Person-years	230446.1	233960.6	235470.4	236361.3		
Incidence rate per 100 person-years (95% CI)	0.263 (0.242, 0.284)	0.203 (0.186, 0.223)	0.164 (0.148, 0.181)	0.161 (0.145, 0.178)		
Unadjusted	1.00 (reference)	0.77 (0.69, 0.87)	0.62 (0.55, 0.71)	0.61 (0.54, 0.69)	0.98 (0.98, 0.99)	<0.001
Model 1 ^c^	1.00 (reference)	0.79 (0.70, 0.89)	0.64 (0.57, 0.73)	0.65 (0.57, 0.74)	0.98 (0.98, 0.99)	<0.001
Model 2 ^d^	1.00 (reference)	0.85 (0.75, 0.96)	0.69 (0.61, 0.79)	0.71 (0.62, 0.81)	0.98 (0.98, 0.99)	<0.001
Non-small cell lung cancer						
Cases	517	394	336	339		
Person-years	230446.1	233960.6	235470.4	236361.3		
Incidence rate per 100 person-years (95% CI)	0.224 (0.206, 0.245)	0.168 (0.153, 0.186)	0.143 (0.128, 0.159)	0.143 (0.129, 0.160)		
Unadjusted	1.00 (reference)	0.75 (0.66, 0.85)	0.63 (0.55, 0.73)	0.64 (0.56, 0.73)	0.98 (0.98, 0.99)	<0.001
Model 1 ^c^	1.00 (reference)	0.76 (0.67, 0.87)	0.65 (0.57, 0.75)	0.67 (0.58, 0.77)	0.98 (0.98, 0.99)	<0.001
Model 2 ^d^	1.00 (reference)	0.82 (0.72, 0.93)	0.70 (0.61, 0.80)	0.73 (0.63, 0.84)	0.99 (0.98, 0.99)	<0.001
Small cell lung cancer						
Cases	88	82	49	41		
Person-years	230446.1	233960.6	235470.4	236361.3		
Incidence rate per 100 person-years (95% CI)	0.038 (0.031, 0.047)	0.035 (0.028, 0.043)	0.021 (0.016, 0.028)	0.017 (0.013, 0.024)		
Unadjusted	1.00 (reference)	0.92 (0.68, 1.24)	0.54 (0.38, 0.77)	0.45 (0.31, 0.66)	0.97 (0.96, 0.98)	<0.001
Model 1 ^c^	1.00 (reference)	0.96 (0.71, 1.30)	0.59 (0.41, 0.84)	0.52 (0.36, 0.76)	0.98 (0.96, 0.99)	<0.001
Model 2 ^d^	1.00 (reference)	1.04 (0.77, 1.41)	0.64 (0.45, 0.92)	0.59 (0.40, 0.85)	0.98 (0.97, 0.99)	<0.001

HR, hazard ratio; CI, confidence interval. ^a^Trend test was performed using median value of each diet score quintile as a continuous variable. ^b^Including 1846 lung cancer cases which has 1,586 non-small cell lung cancer cases and 260 small cell lung cancer cases. ^c^Model 1 was controlled with age (continuous), sex (male, female), race (white, non-white), education levels (college below, some college and college graduate, postgraduate), marital (married, unmarried). ^d^Model 2 was additionally controlled with family history of lung cancer (no, yes, unknown), BMI at baseline (continuous), smoking status (never, current/former), alcohol drinking status (never, current/former, unknown), emphysema history (no, yes), chronic bronchitis history (no, yes), history of hypertension (no, yes), aspirin use (no, yes), history of diabetes (no, yes).

### 3.3 Additional analyses

**Figure 3:**
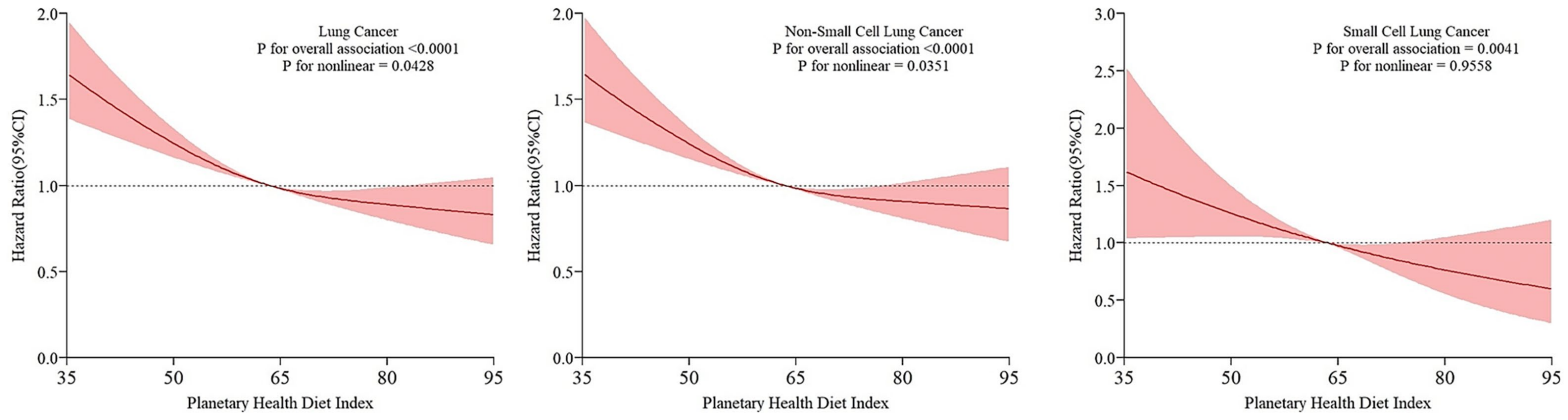
RCS model on the association of Planetary Health Diet Index with the lung cancer incidence. Hazard ratio was adjusted for age (continuous), sex (male, female), race (white, non-white), education levels (college below, some college and college graduate, postgraduate), marital (married, unmarried), family history of lung cancer (no, yes, unknown), BMI at baseline (continuous), smoking status (never, current/former), alcohol drinking status (never, current/former, unknown), emphysema history (no, yes), chronic bronchitis history (no, yes), history of hypertension (no, yes), aspirin use (no, yes), history of diabetes (no, yes).

The association between the PHDI and the risk of lung cancer and its subtypes was characterized using restricted cubic spline plots (Figure 3), which exhibited a non-linear inverse dose–response pattern for lung cancer and NSCLC (P _non-linear_: 0.043 for lung cancer; 0.035 for NSCLC), and a linear inverse dose–response pattern for SCLC (P _non-linear_: 0.956). Subgroup analyses revealed no evidence of interaction by factors such as demographics, personal habits, or related medical history (Table 3; all P _interaction_ > 0.05). We further conducted a sensitivity analysis that excluded participants who had a history of emphysema, chronic bronchitis, or a family history of lung cancer; those with extreme BMI or energy intake values; and incident cases identified within the first 1–2 years of follow-up. In this analysis, the inverse associations remained statistically significant (Table 4; all P _trend_ < 0.001).

**Table 3:** Subgroup analyses on the association between planetary health diet index and lung cancer incidence.

Subgroup variable	Cases	Person-years	Planetary Health Diet Index, HR (95% CI)^c^				*P*_trend_^a^	*P*_interaction_^b^
			Quartile 1	Quartile 2	Quartile 3	Quartile 4		
Age (years)								0.981
≤65	1,065	660858.5	1.00 (reference)	0.86 (0.74, 1.01)	0.71 (0.60, 0.84)	0.73 (0.61, 0.87)	<0.001	
>65	781	275380.0	1.00 (reference)	0.86 (0.71, 1.04)	0.70 (0.57, 0.85)	0.72 (0.59, 0.88)	<0.001	
Sex								0.948
Male	1,073	443121.6	1.00 (reference)	0.83 (0.71, 0.97)	0.69 (0.58, 0.82)	0.70 (0.59, 0.83)	<0.001	
Female	773	493116.9	1.00 (reference)	0.88 (0.72, 1.06)	0.70 (0.57, 0.86)	0.72 (0.58, 0.88)	<0.001	
Race								0.748
White	1710	854521.6	1.00 (reference)	0.84 (0.74, 0.95)	0.69 (0.61, 0.79)	0.71 (0.62, 0.81)	<0.001	
Non-white	136	81716.9	1.00 (reference)	1.09 (0.66, 1.81)	0.73 (0.44, 1.23)	0.73 (0.45, 1.18)	0.091	
Education levels								0.337
College below	951	391563.2	1.00 (reference)	0.78 (0.66, 0.92)	0.66 (0.55, 0.79)	0.78 (0.65, 0.93)	<0.001	
Some college and college graduate	701	366399.2	1.00 (reference)	0.96 (0.79, 1.17)	0.75 (0.61, 0.93)	0.66 (0.53, 0.82)	<0.001	
Postgraduate	194	178276.1	1.00 (reference)	0.80 (0.53, 1.21)	0.68 (0.45, 1.03)	0.65 (0.44, 0.97)	0.033	
Marital								0.597
Married	1,348	737346.4	1.00 (reference)	0.82 (0.71, 0.94)	0.69 (0.60, 0.81)	0.72 (0.62, 0.84)	<0.001	
Unmarried	498	198892.1	1.00 (reference)	0.93 (0.74, 1.18)	0.69 (0.54, 0.89)	0.67 (0.52, 0.86)	<0.001	
Smoking status								0.054
Never	155	459001.7	1.00 (reference)	0.96 (0.59, 1.55)	1.03 (0.65, 1.65)	1.08 (0.68, 1.73)	0.658	
Current/Former	1,691	477236.8	1.00 (reference)	0.85 (0.75, 0.96)	0.67 (0.59, 0.77)	0.69 (0.60, 0.79)	<0.001	
Alcohol drinking status								0.480
Never	75	96176.7	1.00 (reference)	0.79 (0.41, 1.53)	0.58 (0.29, 1.16)	0.98 (0.53, 1.82)	0.883	
Former/Current	1724	814082.2	1.00 (reference)	0.85 (0.75, 0.96)	0.69 (0.61, 0.79)	0.70 (0.61, 0.80)	<0.001	
Unknown	47	25979.7	1.00 (reference)	0.77 (0.34, 1.74)	0.96 (0.45, 2.05)	0.60 (0.25, 1.40)	0.312	
Family history of lung cancer								0.422
No	1,457	817436.6	1.00 (reference)	0.81 (0.70, 0.92)	0.65 (0.57, 0.76)	0.67 (0.57, 0.77)	<0.001	
Yes	317	97038.2	1.00 (reference)	1.03 (0.77, 1.38)	0.84 (0.61, 1.15)	0.84 (0.61, 1.16)	0.185	
Unknown	72	21763.7	1.00 (reference)	0.90 (0.48, 1.70)	0.97 (0.51, 1.85)	1.11 (0.57, 2.14)	0.769	
Emphysema history								0.358
No	1,659	918702.4	1.00 (reference)	0.83 (0.73, 0.94)	0.67 (0.58, 0.76)	0.70 (0.61, 0.80)	<0.001	
Yes	187	17536.1	1.00 (reference)	1.05 (0.73, 1.51)	0.97 (0.66, 1.43)	0.75 (0.47, 1.21)	0.321	
Chronic bronchitis history								0.506
No	1,679	897729.6	1.00 (reference)	0.86 (0.76, 0.98)	0.69 (0.60, 0.79)	0.72 (0.63, 0.83)	<0.001	
Yes	167	38508.9	1.00 (reference)	0.75 (0.51, 1.10)	0.75 (0.50, 1.12)	0.55 (0.34, 0.91)	0.015	
History of diabetes								0.975
No	1719	877655.2	1.00 (reference)	0.84 (0.74, 0.96)	0.69 (0.61, 0.79)	0.71 (0.62, 0.81)	<0.001	
Yes	127	58583.3	1.00 (reference)	0.90 (0.56, 1.44)	0.68 (0.41, 1.13)	0.69 (0.42, 1.14)	0.095	
BMI at baseline (kg/m^2^)								0.416
≤25	714	326801.5	1.00 (reference)	0.80 (0.66, 0.97)	0.61 (0.50, 0.75)	0.62 (0.50, 0.76)	<0.001	
>25	1,132	609437.0	1.00 (reference)	0.87 (0.75, 1.02)	0.75 (0.64, 0.89)	0.78 (0.66, 0.92)	<0.001	
Aspirin use								0.268
No	933	501743.5	1.00 (reference)	0.92 (0.78, 1.09)	0.67 (0.56, 0.81)	0.76 (0.63, 0.91)	<0.001	
Yes	913	434495.0	1.00 (reference)	0.77 (0.65, 0.92)	0.71 (0.59, 0.85)	0.66 (0.54, 0.79)	<0.001	

HR, hazard ratio; CI, confidence interval. ^a^Trend test was performed using median value of each diet score quintile as a continuous variable. ^b^*p* value for interaction was estimated using the likelihood ratio test comparing the model with and without the interaction term of the PHDI and the respective stratification variable. ^c^Hazard ratios were adjusted for age (continuous), sex (male, female), race (white, non-white), education levels (college below, some college and college graduate, postgraduate), marital (married, unmarried), family history of lung cancers (no, yes, unknown), BMI at baseline (continuous), smoking status (never, current/former), alcohol drinking status (never, former/current, unknown), emphysema history (no, yes), chronic bronchitis history (no, yes), history of hypertension (no, yes), history of diabetes (no, yes), aspirin use (no, yes).

**Table 4:** Sensitivity analyses on the association between planetary health diet index and lung cancer incidence.

Categories	Participants	Cases	HR quartile 4 vs. Quartile 1 (95% CI) ^a^	P_-trend_
Primary analysis	106,542	1846	0.71 (0.63, 0.81)	<0.001
Excluding participants with emphysema history	104,295	1,659	0.71 (0.62, 0.81)	<0.001
Excluding participants with chronic bronchitis history	101,969	1,679	0.73 (0.64, 0.84)	<0.001
Excluding participants with extreme BMI (baseline)^b^	104,373	1805	0.72 (0.63, 0.83)	<0.001
Excluding participants with extreme energy intake^c^	105,037	1804	0.73 (0.64, 0.83)	<0.001
Excluding cases observed within the first 1 year of follow-up	106,372	1,676	0.70 (0.61, 0.80)	<0.001
Excluding cases observed within the first 2 year of follow-up	106,217	1,521	0.68 (0.58, 0.78)	<0.001
Excluded participants with family history of lung cancer	92,849	1,457	0.67 (0.58, 0.78)	<0.001

^a^Hazard ratios were adjusted for age (continuous), sex (male, female), race (white, non-white), education levels (college below, some college and college graduate, postgraduate), marital (married, unmarried), family history of lung cancers (no, yes, unknown), BMI at baseline (≤25, >25), smoking status (never, current/former), alcohol drinking status (never, former/current, unknown), emphysema history (no, yes), chronic bronchitis history (no, yes), history of hypertension (no, yes), history of diabetes (no, yes), aspirin use (no, yes). ^b^The extreme BMI (baseline) was defined as top 1% or bottom 1% in the included population; ^c^The extreme energy intake was defined as >4,000 kcal/day or <500 kcal/day.

### 3.4 Individual components and the risk of lung cancer

To assess the contribution of specific dietary aspects, the associations of the four distinct PHDI components with lung cancer incidence were evaluated. As shown on Supplementary Tables 2–5, the incidence of lung cancer reduced with higher scores of adequacy component (HR _Quartile 4 vs Quartile 1_: 0.64; 95% CI: 0.56, 0.74; P _trend_ < 0.001) and ratio component (HR _Quartile 4 vs Quartile 1_: 0.71; 95% CI: 0.61, 0.82; P _trend_ < 0.001). No significant association was observed between the risk of lung cancer and the score of the optimum component (HR _Quartile 4 vs Quartile 1_: 1.00; 95% CI: 0.88, 1.14; P _trend_: 0.802) and that of the moderation component (HR _Quartile 4 vs Quartile 1_: 1.01; 95% CI: 0.88, 1.15; P _trend_: 0.968).

## 4 Discussion

In this large-scale cohort analysis based on the PLCO database, our findings indicated that strict adherence to the PHD was significantly associated with a reduced risk of lung cancer and its various subtypes (NSCLC and SCLC). The restricted cubic spline plot indicated a continuously declining trend of the risk of lung cancer and its subtypes as PHDI rose. Among these, PHDI had a non-linear association with lung cancer and NSCLC. However, it exhibited a linear dose–response relationship with SCLC. In the subgroup analysis, we did not find any significant interaction. Furthermore, several sensitivity analyses were conducted, which consistently indicated no substantial alterations in the results, thereby providing additional support for our conclusions.

The PHD was proposed by the EAT-Lancet Commission, aiming to promote both human well-being and environmental sustainability (7). The core premise of this dietary pattern, which lies in its integration of human health with environmental sustainability, has established it as a prominent research focus. Research conducted on Singaporean Chinese populations revealed a significant negative correlation between elevated PHD compliance levels and various mortality risks, including deaths from all causes, cardiovascular conditions, malignancies, and respiratory illnesses (14). Another meta-analysis also found that greater adherence to the PHD showed inverse associations with all-cause mortality (15). However, the available evidence on the link between PHD and lung cancer susceptibility is still sparse and inconsistent, which hinders the formation of a definitive consensus. For instance, evidence from a prospective cohort study linking higher PHD adherence to reduced lung cancer incidence (8) stands in contrast to findings that suggest a significant association only in men, pointing to potential sex-based discrepancies (9). Our research aims to use another evaluation standard, the PHDI to verify this association. In contrast to the previous studies, the PHDI, by incorporating dietary energy content, enables a more accurate assessment of individuals’ adherence. Besides, PHDI provided a more detailed classification for the scores of vegetables consumption, and took into account most of the intermediate values and interchangeable groups proposed in EAT-Lancet. Last, some confounding factors of related medical history, such as emphysema history and chronic bronchitis history, were not considered in previous studies. As a result, our study filled the knowledge gap by fully taking into account these confounding factors and further exploring the relationship between PHDI and lung cancer.

After further studying the four different components of PHDI, we found that moderately increasing the intake of the adequacy component was associated with a reduced risk of lung cancer. This indicated that the foods comprising this adequacy component, including nuts, legumes, fruits, vegetables and grains may be protective factors for lung cancer. While previous studies have established the benefits of these individual food in isolation (4, 16–18), our findings provide novel evidence that these protective effects persist and contribute to risk reduction even within the complex context of an overall dietary pattern like the PHD. In addition, we found that ensuring the ratio component within an appropriate range also had a significant relationship with a reduced risk of lung cancer. Though no other studies have proved this potential association, according to Eat-Lancet, it suggested that the greatest benefit may be derived from a diverse intake of vegetables (7). Interestingly, in our study, the optimum and moderation components did not show a significant relationship with lung cancer risk. Regarding specific food items, our findings on eggs, fish, and poultry align with previous research that evaluated these items individually (19–21). To illustrate, a study based on IROPICAN in Iran found no significant correlation between the diet rich in eggs consumption and the lung cancer susceptibility (20). Others showed that the intake of total poultry and fish was not related to the lung cancer susceptibility (19, 21). As for other dietary components, the situation is more complicated. For example, while a pooled analysis of multicenter case–control study linked daily potato consumption to elevated lung cancer risk (22), our study did not find this association within the PHDI framework. This suggests that the potential risk of specific foods might be mitigated when consumed as part of an overall diet. In addition, there is a lack of relevant research on the impact of vegetable oils, animal fats and added sugar consumption on lung cancer, and the influence of dairy on lung cancer remains controversial (23, 24). For red meat, on the basis of evidence primarily concerning colorectal cancer, the International Agency for Research on Cancer (IARC) has classified processed red meat as a Group 1 carcinogen (carcinogenic to humans); due to less consistent data, unprocessed red meat was classified as Group 2 (probably carcinogenic to humans) (7). But the evidence on unprocessed red meat related to lung cancer was still ambiguous (2, 20, 25). Given the complexity of dietary interactions, the overall dietary pattern may be a more effective predictor of lung cancer risk than the intake of a single food. Further research is needed to explore the interaction between specific foods and the overall dietary pattern.

The underlying mechanism by which PHDI reduced the risk of lung cancer may stem from the cumulative and synergistic effects of the overall dietary pattern. Primarily, the PHDI represents an overall dietary pattern characterized by a high density of phytochemicals, including isothiocyanates, indoles, and flavonoids. These compounds likely exert additive protective effects by inducing apoptosis and exerting antioxidant and anti-inflammatory effects (26–28). Furthermore, the PHDI advocates for limited consumption of processed red meat, which reduces exposure to carcinogens inherent, such as nitrate, nitrite, and other preservatives. By simultaneously reducing exposure to carcinogens and providing substrates to counteract oxidative stress, the PHDI creates a systemic environment less conducive to tumorigenesis. Second, the PHDI recommended a balanced variety of different-colored vegetables, which may help in obtaining a more comprehensive range of phytochemicals and nutrients and could potentially reduce the risk of cancer (29). Additionally, the dietary pattern’s efficacy in preventing obesity (30) serves as a downstream mediator, further lowering lung cancer risk (31). However, the current evidence remains limited, and further studies are warranted to confirm this association.

Interestingly, our restricted cubic spline analysis revealed distinct dose–response patterns: a non-linear inverse association was observed for NSCLC, whereas a linear inverse association was observed for SCLC. This divergence may be explained by the distinct pathophysiological mechanisms and heterogeneity of these subtypes. Specifically, the risk of NSCLC declined sharply with increasing PHDI scores before plateauing at higher levels of adherence. This non-linearity suggests a saturation effect. Individuals with low PHDI scores typically endure chronic high oxidative stress, elevated inflammatory burden, and increased exposure to carcinogens. Consequently, initial improvements in diet quality can substantially mitigate these pathophysiological processes, leading to a marked reduction in disease risk. However, once the PHDI reaches a relatively high level, some key oncogenic pathways (such as oxidative stress and inflammation) have reached a considerable degree of regulation. Given the high heterogeneity of NSCLC and the diversity of its pathogenic signaling pathways (32), further increases in PHDI offers limited protective effect in combating other carcinogenic mechanisms, thereby leading to the plateau effect. In contrast, SCLC exhibits less molecular heterogeneity and is driven by more uniform oncogenic mechanisms (33). Consequently, the regulatory effect of dietary factors does not appear to exhibit a significant saturation effect, thereby maintaining a linear inverse association.

Our research has the following advantages. Most importantly, a novel aspect of this study is the use of an energy-adjusted scoring method which is more applicable to various calorie intake scenarios. In addition, our research is a prospective study based on a large population with a long follow-up period, which makes our study reliable. Besides, our study was methodologically rigorous as we fully took into account various confounding factors and conducted subgroup analysis and sensitivity analysis.

However, there are several limitations in our study. First, in our study, we did not observe a significant relationship between some potential risk factors and lung cancer, such as red meat. This might be due to the fact that the threshold value was too low, and most people could not reach it, resulting in an unclear distinction in the scores. Second, dietary exposure was assessed via self-reported questionnaires, which is subject to measurement error and recall bias. Furthermore, dietary habits may have changed during the 8.8-year follow-up period, while we only relied on baseline diet. Although the DHQ is a validated instrument and well-established dietary habits often exhibit relative stability in old people (34), this single assessment may not capture long-term adherence perfectly. Third, it is important to note that the PHDI used in this study is based on the Institute of Health Metrics and Evaluation’s Theoretical Minimal Risk Exposure Level (TMREL) Methodology, where TMREL values are primarily derived from systematic reviews of the medical literature. Current evidence indicates that approximately 80–90% of high-quality systematic reviews on diet and health outcomes are based on data from high-income countries. Therefore, the PHDI may not be directly applicable to low- and middle-income country populations. Coupled with the demographic characteristics of the PLCO cohort (predominantly White adults aged 55–74 years), the generalizability of our findings to younger populations and other ethnic or global populations is limited. Given the incomplete consistency between TMREL and the Global Burden of Disease (GBD) data modeling (35), future studies are needed to enrich and validate the global evidence on the associations between this dietary pattern and health. Fourth, while our study showed protective effects for adequacy and ratio components, no significant associations for moderation or optimum components were found. This suggests that the composite index may mask nuanced dietary effects. Further research is needed to explore the effects of individual dietary components and the interaction between specific foods and the overall dietary pattern. Fifth, the selection of confounding factors was informed by existing literature and clinical expertise. The possibility of over-adjustment of covariates, which might influence our findings, cannot be ruled out. Sixth, while PHD emphasizes both human health and environmental sustainability, our study only focus on the lung cancer. Further researchers are needed to explore the effects of the PHD on both human health and the environment. Finally, as an observational cohort study, our design precludes the establishment of causality. Although we have rigorously adjusted for major confounders, residual confounding (particularly regarding the intensity of smoking or occupational exposures) may still influence the observed associations. Therefore, our findings should be interpreted as robust epidemiological evidence supporting the potentially protective effects of the PHDI, rather than definitive proof of causation.

## 5 Conclusion

Among the American population aged 55 to 74, higher PHDI was related to the lower risk of lung cancer and its subtypes. More research is needed to verify whether such a connection exists in other populations and regions.

## Data Availability

The original contributions presented in the study are included in the article/Supplementary material, further inquiries can be directed to the corresponding author/s.
