# Supplementary material for: Association between planetary health diet index and lung cancer risk in 106,542 participants: a prospective cohort study
Source: Front Nutr. 2026 Apr 29;13:1794585. doi: 10.3389/fnut.2026.1794585 (PMC13167498; doi:10.3389/fnut.2026.1794585)
Supplement: Supplementary file 1 [file Table_1.DOCX]

**SUPPLEMENTARY MATERIAL**

**Association Between Planetary Health Diet Index and Lung Cancer Risk in 106542 Participants: A Prospective Cohort Study**

Table of contents

**Supplementary Table 1**. Criteria for determining the Planetary Health Diet Index.

**Supplementary Table 2**. Hazard ratios of the association of Adequacy component with the risk of lung cancer.

**Supplementary Table 3**. Hazard ratios of the association of Optimum component with the risk of lung cancer.

**Supplementary Table 4**. Hazard ratios of the association of Ratio component with the risk of lung cancer.

**Supplementary Table 5**. Hazard ratios of the association of Moderation component with the risk of lung cancer.

**Supplementary Table 1.** Criteria for determining the Planetary Health Diet Index.

| **Food components of PHDI^1^** | Target intake (reference interval) ^2^ | 0 points | 5 points | 10 points | 5 points | 0 points |
| --- | --- | --- | --- | --- | --- | --- |
| **Adequacy component** |  |  |  |  |  |  |
| Nuts and peanuts | ≥11.6 | 0.0 |  | ≥11.6 |  |  |
| Legumes | ≥11.3 | 0.0 |  | ≥11.3 |  |  |
| Fruits | ≥5.0 | 0.0 |  | ≥5.0 |  |  |
| Vegetables | ≥3.1 | 0.0 |  | ≥3.1 |  |  |
| Whole grains | ≥32.4 | 0.0 |  | ≥32.4 |  |  |
| **Optimum component** |  |  |  |  |  |  |
| Eggs | 0.8 | 0.0 |  | 0.8 |  | ≥1.5 |
| Fish and seafood | 1.6 | 0.0 |  | 1.6 |  | ≥5.7 |
| Potatoes | 1.6 | 0.0 |  | 1.6 |  | ≥3.1 |
| Dairy | 6.1 | 0.0 |  | 6.1 |  | ≥12.2 |
| Vegetable oils | 16.5 | 0.0 |  | 16.5 |  | ≥30.7 |
| **Ratio component** |  |  |  |  |  |  |
| DGV^2^ | 29.5 | 0.0 | 29.5 |  | 29.5 | 100 |
| ReV^3^ | 38.5 | 0.0 | 38.5 |  | 38.5 | 100 |
| **Moderation component** |  |  |  |  |  |  |
| Red meat | 0.0 | ≥2.4 |  | 0.0 |  |  |
| Chicken | 0.0 | ≥5.0 |  | 0.0 |  |  |
| Animal fats | 0.0 | ≥1.4 |  | 0.0 |  |  |
| Added sugars | 0.0 | ≥4.8 |  | 0.0 |  |  |

**1** Food components in the index are based on the Planetary Health Diet as caloric densities.

**2** Dark green to total vegetables ratio

**3** Red and orange to total vegetables ratio

**Supplementary Table 2.** Hazard ratios of the association of Adequacy component with the risk of lung cancer **^a^**

| **Quartiles of adequacy component score** | **Number of subjects/cases** | **Person-years** | **Incidence rate per 100 person-years (95% confidence interval)** | **Hazard ratio (95% confidence interval)** | | | | |
| --- | --- | --- | --- | --- | --- | --- | --- | --- |
|  |  |  |  | **Unadjusted** | **Model 1^b^** | **Model 2^c^** | | **Model 3^d^** |
| Quartile 1 (≤20.80) | 26668/688 | 230200.154 | 0.299 (0.277, 0.322) | 1.00 (reference) | 1.00 (reference) | | 1.00 (reference) | 1.00 (reference) |
| Quartile 2 (20.81-24.62) | 26645/467 | 235386.029 | 0.198 (0.181, 0.217) | 0.66 (0.59, 0.74) | 0.69 (0.61, 0.77) | | 0.78 (0.69, 0.88) | 0.81 (0.72, 0.91) |
| Quartile 3 (24.63-28.67) | 26573/366 | 235417.588 | 0.155 (0.14, 0.172) | 0.52 (0.46, 0.59) | 0.54 (0.47, 0.61) | | 0.64 (0.56, 0.72) | 0.68 (0.59, 0.77) |
| Quartile 4 (>28.67) | 26656/325 | 235234.731 | 0.138 (0.124, 0.154) | 0.46 (0.40, 0.53) | 0.48 (0.42, 0.55) | | 0.59 (0.51, 0.67) | 0.64 (0.56, 0.74) |
| *P* for trend |  |  |  | <0.001 | <0.001 | | <0.001 | <0.001 |

**a** Values are given as HRs and 95% CIs within parentheses, with the respective 0-point group as the reference group. Multivariate proportional hazards were used to examine the associations.

**b**: Model 1 was controlled with age (continuous), sex (male, female), race (white, non-white), education levels (college below, some college and college graduate, postgraduate), marital (married, unmarried).

**c**: Model 2 was additionally controlled on the Model 1 with family history of lung cancer (no, yes, unknown), BMI at baseline (continuous), smoking status (never, current/former), alcohol drinking status (never, current/former, unknown), emphysema history (no, yes), chronic bronchitis history (no, yes), history of hypertension (no, yes), aspirin use(no, yes), history of diabetes(no, yes).

**d**: Model 3 was additionally controlled on the Model 2 with the score of Optimum component (continuous), Ratio component (continuous) and Moderation component (continuous).

**Supplementary Table 3.** Hazard ratios of the association of Optimum component with the risk of lung cancer **^a^**

| **Quartiles of optimum component score** | **Number of subjects/cases** | **Person-years** | **Incidence rate per 100 person-years (95% confidence interval)** | **Hazard ratio (95% confidence interval)** | | | | |
| --- | --- | --- | --- | --- | --- | --- | --- | --- |
|  |  |  |  | **Unadjusted** | **Model 1^b^** | **Model 2^c^** | | **Model 3^d^** |
| Quartile 1 (≤18.71) | 26622/507 | 231155.649 | 0.219 (0.201, 0.239) | 1.00 (reference) | 1.00 (reference) | | 1.00 (reference) | 1.00 (reference) |
| Quartile 2 (18.72-23.51) | 26622/437 | 233787.977 | 0.187 (0.17, 0.205) | 0.85 (0.75, 0.97) | 0.87 (0.77, 0.99) | | 0.89 (0.78, 1.01) | 0.92 (0.81, 1.05) |
| Quartile 3 (23.52-28.36) | 26675/455 | 235873.624 | 0.193 (0.176, 0.211) | 0.88 (0.77, 1.00) | 0.92 (0.81, 1.05) | | 0.94 (0.82, 1.06) | 0.99 (0.87, 1.13) |
| Quartile 4 (>28.36) | 26623/447 | 235421.251 | 0.19 (0.173, 0.208) | 0.86 (0.76, 0.98) | 0.92 (0.81, 1.05) | | 0.92 (0.81, 1.05) | 1.00 (0.88, 1.14) |
| *P* for trend |  |  |  | 0.038 | 0.302 | | 0.303 | 0.802 |

**a** Values are given as HRs and 95% CIs within parentheses, with the respective 0-point group as the reference group. Multivariate proportional hazards were used to examine the associations.

**b**: Model 1 was controlled with age (continuous), sex (male, female), race (white, non-white), education levels (college below, some college and college graduate, postgraduate), marital (married, unmarried).

**c**: Model 2 was additionally controlled on the Model 1 with family history of lung cancer (no, yes, unknown), BMI at baseline (continuous), smoking status (never, current/former), alcohol drinking status (never, current/former, unknown), emphysema history (no, yes), chronic bronchitis history (no, yes), history of hypertension (no, yes), aspirin use(no, yes), history of diabetes(no, yes).

**d**: Model 3 was additionally controlled on the Model 2 with the score of Adequacy component (continuous), Ratio component (continuous) and Moderation component (continuous).

**Supplementary Table 4.** Hazard ratios of the association of Ratio component with the risk of lung cancer **^a^**

| **Quartiles of ratio component score** | **Number of subjects/cases** | **Person-years** | **Incidence rate per 100 person-years (95% confidence interval)** | **Hazard ratio (95% confidence interval)** | | | | |
| --- | --- | --- | --- | --- | --- | --- | --- | --- |
|  |  |  |  | **Unadjusted** | **Model 1^b^** | **Model 2^c^** | | **Model 3^d^** |
| Quartile 1 (≤1.19) | 26545/641 | 229499.045 | 0.279 (0.259, 0.302) | 1.00 (reference) | 1.00 (reference) | | 1.00 (reference) | 1.00 (reference) |
| Quartile 2 (1.20-1.90) | 26862/502 | 235756.294 | 0.213 (0.195, 0.232) | 0.76 (0.68, 0.86) | 0.82 (0.73, 0.92) | | 0.87 (0.78, 0.98) | 0.93 (0.83, 1.05) |
| Quartile 3 (1.91-2.96) | 26467/390 | 234002.595 | 0.167 (0.151, 0.184) | 0.60 (0.52, 0.68) | 0.68 (0.60, 0.78) | | 0.74 (0.65, 0.84) | 0.81 (0.71, 0.93) |
| Quartile 4 (>2.96) | 26668/313 | 236980.567 | 0.132 (0.118, 0.148) | 0.47 (0.41, 0.54) | 0.56 (0.49, 0.65) | | 0.61 (0.53, 0.71) | 0.71 (0.61, 0.82) |
| *P* for trend |  |  |  | <0.001 | <0.001 | | <0.001 | <0.001 |

**a** Values are given as HRs and 95% CIs within parentheses, with the respective 0-point group as the reference group. Multivariate proportional hazards were used to examine the associations.

**b**: Model 1 was controlled with age (continuous), sex (male, female), race (white, non-white), education levels (college below, some college and college graduate, postgraduate), marital (married, unmarried).

**c**: Model 2 was additionally controlled on the Model 1 with family history of lung cancer (no, yes, unknown), BMI at baseline (continuous), smoking status (never, current/former), alcohol drinking status (never, current/former, unknown), emphysema history (no, yes), chronic bronchitis history (no, yes), history of hypertension (no, yes), aspirin use(no, yes), history of diabetes(no, yes).

**d**: Model 3 was additionally controlled on the Model 2 with the score of Adequacy component (continuous), Optimum component (continuous) and Moderation component (continuous).

**Supplementary Table 5.** Hazard ratios of the association of Moderation component with the risk of lung cancer **^a^**

| **Quartiles of Moderation component score** | **Number of subjects/cases** | **Person-years** | **Incidence rate per 100 person-years (95% confidence interval)** | **Hazard ratio (95% confidence interval)** | | | | |
| --- | --- | --- | --- | --- | --- | --- | --- | --- |
|  |  |  |  | **Unadjusted** | **Model 1^b^** | **Model 2^c^** | | **Model 3^d^** |
| Quartile 1 (≤8.32) | 26647/426 | 233815.193 | 0.182 (0.166, 0.2) | 1.00 (reference) | 1.00 (reference) | | 1.00 (reference) | 1.00 (reference) |
| Quartile 2 (8.33-12.89) | 26625/487 | 234280.309 | 0.208 (0.19, 0.227) | 1.14 (1.00, 1.30) | 1.09 (0.96, 1.24) | | 1.06 (0.93, 1.21) | 1.07 (0.94, 1.22) |
| Quartile 3 (12.90-17.68) | 26649/485 | 234265.475 | 0.207 (0.189, 0.226) | 1.14 (1.00, 1.29) | 1.08 (0.95, 1.23) | | 1.04 (0.91, 1.18) | 1.07 (0.94, 1.22) |
| Quartile 4 (>17.68) | 26621/448 | 233877.524 | 0.192 (0.175, 0.21) | 1.05 (0.92, 1.20) | 0.99 (0.87, 1.13) | | 0.95 (0.83, 1.08) | 1.01 (0.88, 1.15) |
| *P* for trend |  |  |  | 0.647 | 0.742 | | 0.286 | 0.968 |

**a** Values are given as HRs and 95% CIs within parentheses, with the respective 0-point group as the reference group. Multivariate proportional hazards were used to examine the associations.

**b**: Model 1 was controlled with age (continuous), sex (male, female), race (white, non-white), education levels (college below, some college and college graduate, postgraduate), marital (married, unmarried).

**c**: Model 2 was additionally controlled on the Model 1 with family history of lung cancer (no, yes, unknown), BMI at baseline (continuous), smoking status (never, current/former), alcohol drinking status (never, current/former, unknown), emphysema history (no, yes), chronic bronchitis history (no, yes), history of hypertension (no, yes), aspirin use(no, yes), history of diabetes(no, yes).

**d**: Model 3 was additionally controlled on the Model 2 with the score of Adequacy component (continuous), Optimum component (continuous) and Ratio component (continuous).
